# ‘A group of totally awesome people who do stuff’ - a qualitative descriptive study of a children and young people’s patient and public involvement endeavour

**DOI:** 10.1186/s40900-019-0148-0

**Published:** 2019-03-12

**Authors:** Faye Forsyth, Caroline Saunders, Anne Elmer, Shirlene Badger

**Affiliations:** 1 Cambridge Clinical Research Centre, Addenbrooke’s Hospital, Cambridge, UK; 2 Institute of Public Health University of Cambridge, Cambridge, UK

**Keywords:** Patient and public involvement, Children and young people, Qualitative description

## Abstract

**Background:**

In 2013, the Cambridge Clinical Research Facility (CCRF) set up a Children’s Non-Executive Research Board to advise on service and facility development and research involving children and young people (CYP). In 2015, the Children’s Experiences of Engaging in Research study (CHEER) was conceived to explore the Children’s Board as a patient and public involvement initiative.

**Aim:**

To explore the views of CYP, staff and parents involved in the Children’s Board with the view to describe their experiences of the selected mechanism of involvement (Children’s Board) within the context of operation (CCRF).

**Methods:**

A qualitative descriptive methodology involving qualitative content analysis of semi-structured interviews was used to derive descriptive summaries of the interview data.

**Setting and participants:**

Interviews were carried out with staff (*n* = 5), children (*n* = 2) and parents (n = 2) who participated in the first or second Children’s Board meetings.

**Results:**

Twelve descriptive summaries emerged: (1) CCRF ‘role’ perspective (2) purpose, remit and future direction (3) aspirations (4) learning as reciprocation (5) regular meetings, contact and feedback (6) expectation setting and ground rules (7) culture of PPI (8) surprise, underestimation and self-selection (9) reciprocity, incentivisation and participation (10) practicalities, timing and barriers (11) parental roles (12) event structure. These highlighted the importance of selecting the right mechanism of involvement in relation to context for involvement and the reductive biases adults and healthcare providers may unconsciously hold. Both of these aspects may affect the efficacy of PPI endeavours with CYP.

**Discussion and conclusions:**

Mechanisms by which CYP are involved in research should be considered from the outset; taking into consideration both the setting and contextual features. Contextual and process factors important in the adult PPI realm were generally observed in this PPI initiative with CYP; however further research is required to explore unconscious biases and reductive perceptions in adult facilitators.

## Plain English summary

A core aim of the National Institute for Health Research (NIHR) funded Cambridge Clinical Research Facility (CCRF), a purpose-built environment for both adult and paediatric research, is to undertake patient and public involvement activities (PPI). One of the ways the CCRF tried to meet this aim was by forming a Children’s Non-Executive Research Board (Children’s Board). Following the first meeting, staff, children and young people (CYP) and parents involved were interviewed with the view to explore their experiences. This paper describes the twelve themes from these interviews and links this to other research in the field. Although there are a growing number of reports about PPI efforts with CYP, to our knowledge this is the first published report from a Clinical Research Facility setting. Given there are now 23 CRFs across England, it is important PPI endeavours undertaken in this context are detailed and shared to enable further learning and development.

## Introduction

Patient and public involvement (PPI) in clinical research has rapidly progressed from a relatively new field to a burgeoning speciality thanks in part to the commitment of the National Institute for Health Research (NIHR) and other research funding bodies [1, 2]. The aim of PPI is to improve all aspects of the research process from commissioning through to dissemination and evaluation [3]. The growing emphasis of PPI in research has resulted in numerous publications that chart its evolution. Early papers focussed on rationale and methods [4]; in the more recent past attention turned to contextual and process factors important in driving successful PPI [5, 6]; the current zeitgeist is measuring impact to generate empirical evidence of value [5, 7, 8]. Publications on PPI with CYP, defined here as a person under 18 years of age as per the United Nations Convention on the Rights of the Child [9], have not kept pace and the literature on effective engagement remains sparse [10]. This seems surprising given the moral right for CYP to be involved in decisions that affect them, has long been enshrined in both international and UK law [11]. Methods and techniques demonstrated to be effective in the adult PPI realm cannot necessarily be translated to PPI with CYP [12] and publications exploring CYP initiatives highlight the need to develop flexible, youth centred approaches [13, 14]. The aim of this report is to add to the developing evidence base, for as Bird et al. [11] rightly point out, collaborations with CYP must be published in order to share good (and not so good) practice.

## Background

As part of the NIHR ‘infrastructure’ arm [15] the Cambridge Clinical Research Facility (CCRF) [16] has a PPI mandate. In January 2013, CYP attending the CCRF to take part in research studies were informally consulted about replacing a broken games console. The enthusiastic response sparked an idea in staff to more actively involve CYP via an advisory group. A formal survey canvassing opinion and collecting contact details of interested parties was then conducted. Thirty-five children expressed an interest so a staff-led organisation group was formed and the CCRF Children’s Non-Executive Research Board founded (referred to herein as the Children’s Board). The first Children’s Board meeting, held on a Saturday morning in February 2013 at the CCRF, was attended by six CYP, ranging in age from five to sixteen years (median age 9). Paediatric trained staff (*n* = 4) with experience of working with CYP designed and facilitated the event. Parents (*n* = 4) were invited to stay if they wished but were not actively involved beyond consent and administrative aspects (travel expenses, contact details and allergy check prior to lunch). Those parents that chose to stay sat at the back of the meeting room separate from the CYP who were seated in a ‘boardroom’ format at the front alongside the staff facilitators. The focus of the first meeting was to obtain the views of CYP on: service development plans; the design of generic facility and research participant information; and the suitability of metabolic measurement equipment for CYP use.

The first Children’s Board was visually recorded and edited to produce a two minute informational DVD. The DVD was shared leading to more widespread coverage and features on local media as well as the NIHR INVOLVE website [17]. During the planning phase for the second board meeting, the idea of a project exploring the Children’s Board as a PPI initiative was developed. The resulting qualitative descriptive study; Children’s Experiences of Engaging in Research (CHEER) was collaboratively devised by CCRF staff (FF, CS, AE) alongside an academic partner (SB). The CHEER study had many objectives however the primary aim was to examine the views of CYP, staff and parents who participated in the board. Ethics approval by National Research Ethics Service London-Chelsea was granted in October 2015 following proportionate review.

## Definition

Nomenclature remains an issue in PPI and most studies refer to the definition of PPI employed. The CCRF subscribe to the INVOLVE [3] definition of PPI: research “carried out ‘with’ or ‘by’ members of the public rather than ‘to’, ‘about’ or ‘for’ them”. Terminology wise, the Children’s Board could be viewed equivalent to a ‘Young People’s Advisory Group’ (YPAG) [18] or ‘Children’s Advisory Panel’. That is, the Children’s Board is a group of CYP who meet to comment and advise on any aspect of research in the CCRF. Their self-selected tag-line was ‘a group of totally awesome people who do things’.

## Aim

The main aim of the research was to explore the views and experiences of CYP, parents and staff involved in the Children’s Board at the CCRF.

## Methods

Between October 2015 and December 2017, interviews were carried out with nine people. A qualitative descriptive (QD) methodology was employed as it allows for straight forward description, free of the ‘interpretative spin’ conceptual, philosophical or highly abstract frameworks can bring [19]. QD employs content analysis to organize, elicit meaning and draw conclusions from data [20]. Content analysis was selected as it was a good fit given internal (study team experience) and external (resources and time) factors [20]. The approach was inductive and involved coding then grouping excerpts describing the same phenomena to produce descriptive summaries of the data. Coding and initial grouping into categories was performed by FF and cross checked by SB. Emerging summaries were discussed and refined before quotes that 1) best described the informational content of the data and 2) represented the sample, were selected. The resulting descriptive summaries were given a data derived ‘topic title’.

QD has been criticised as elementary or superficial as the resulting interpretation is not highly interpretive, rather ‘low interference’ [19]. Given the small sample size, especially the CYP component, content analysis was accompanied by numerical documentation of the frequencies of statements across the sample subgroups. As this was a mixed cohort, numerical counts proved useful during interpretation and are reported here to demonstrate the internal generalizability of our claims within the sample studied [21]. That is, how characteristic the descriptive summaries are of the collective interviews. This transparent reporting presents readers with the amount of evidence for each of the descriptive summaries and enables them to assess the validity of conclusions. To further strengthen findings, member checking for interpretative validity was performed via a small focus group with two of the staff members whereby the descriptive summaries were presented alongside the quotes collated to demonstrate the theme. Findings are reported in line with the Guidance for Reporting Involvement of Patients and the Public (GRIPP2) checklist [22].

## People involved

**Fig. 1:**
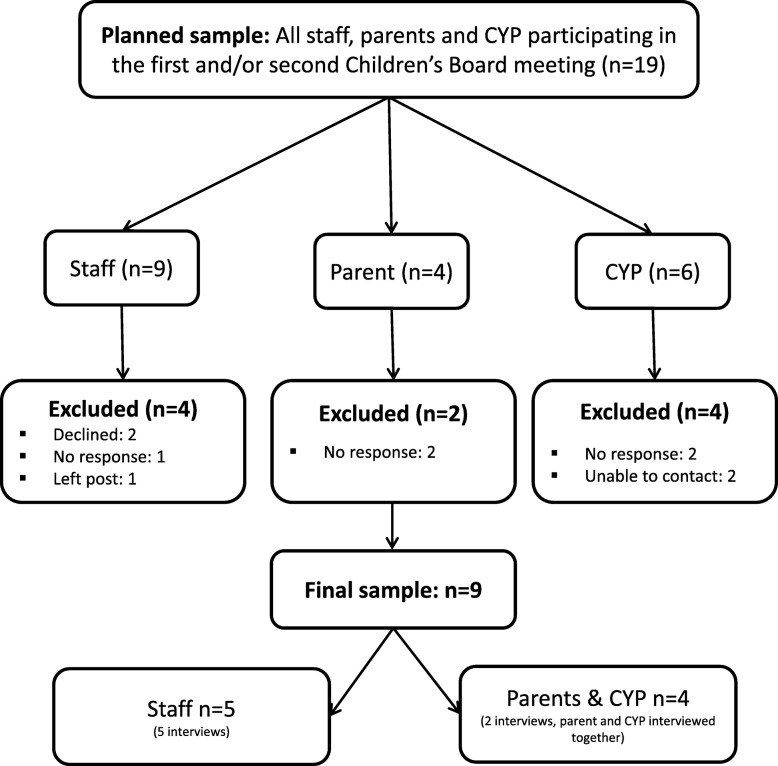
Flow Diagram

Purposeful maximum variation sampling was employed and everyone (*n* = 19) who had participated in the first and/or second Children’s Board meeting were invited to participate (see Fig. 1 Flow Diagram). Five staff (S01 - S05), two parents (P01, P02) and two children (C01, C02) agreed to participate. The initial plan was to interview CYP and parents separately and in the home setting however at their request, all interviews were held at the CCRC. Each participant was provided an age appropriate information sheet and given time to consider participation before informed consent or assent. Demographic data was not collected as it was deemed too intrusive and unnecessary in addressing the aims of the study. Photos from the Children’s Board events were used to stimulate discussion, the CYP were encouraged to take the lead and always addressed first during the combined interviews. Due to a low response rate from CYP Board members, the staff perspective is more extensively represented. As a result, parent and CYP data was used to corroborate or question the descriptive summaries.

## Stages and level of involvement

As the sample size was already small, the Children’s Board members were not directly involved as researchers; rather CYP were involved in shaping the study via protocol and participant information sheet review. The Liverpool YPAG [23] reviewed all documentation including the protocol, information sheet and consent forms. Many recommendations were made such as removing statements in the PIS about ethics committee review, replacing the word signature with write name on the consent form, changing ‘initial box’ on the consent form to circling ‘YES/NO’ statements or happy/sad faces for the youngest ages. All changes recommended were made. The project was also presented at one of the Children’s Board meetings to determine acceptability. On reflection, this project would have been amenable to co-production which could have improved the quality and depth of interview data [11].

## Measurement of PPI impact

Measuring impact was not the primary aim however over the course of the interviews many ‘impact’ examples were given, these are summarized in line with Brett and colleagues categorization [1] (see Table 1). Impacts described tended to be physical. Reference was made to what Brett et al. [1] describe as ‘impacts on users’ like career development and research education; however these were not attributed as ‘impacts’ by interviewees. It is important to acknowledge this is not *all* of the impacts of the Children’s Board, rather a tabulation of aspects described in interviews. If considered to be the end result of the endeavour, then these are undoubtedly tangible impacts. However, a more meaningful measure would be a description of the level of co-production, evidence of use of publications in practice and pre/post room redesign satisfaction questionnaires. In order to have incorporated a more critical review of impact, an evaluation methodology would have had to be conceived at the outset. Previous research has similarly found PPI impacts tend to be ‘brief narrative descriptions’ and ‘biased towards good news’^1^ which has led to calls for more robust measures that indicate the ‘extent or magnitude of impact' [8]. The take home message from this research is early incorporation of an evaluation methodology is key to enable demonstration of impact; especially given this often requires a baseline (pre impact) measure.

**Table 1:** Summary of Impacts

Impact Area	Dimension	Output/Outcome/Product
Research Process	CCRF paediatric healthy volunteer study	Increase in recruitment rate
		Alterations to participant information sheet
		Alterations to protocol (dietary component)
Quality Improvement	CCRF service user co-produced publications	Under 6 Photo Story ‘Ettie takes part in a research study’
		6–10 years Photo Story ‘Lizzie takes part in a research study’
		Eleven+ Photo Story ‘James takes part in a research study’
		Children’s Activity Menu
	CCRF patient experience	Creation of ‘age specific’ patient experience questionnaires
Service Development	CCRF service user co-developed facilities	Design of paediatric phlebotomy room
		Equipment testing for suitability for Paediatric Use (BodPod, Calorimeter Room)
	CCRF service user co-designed website	https://cambridge.CCRF.nihr.ac.uk/patientpublic/involving-children-and-young-people-at-the-CCRF/

## Study results

Twelve descriptive summaries emerged which are separated into contextual or process factors as per the GRIPP2 checklist [22]. Contextual factors: (1) CCRF ‘role’ perspective (2) purpose, remit and future direction (3) aspirations (4) learning as reciprocation (5) regular meetings, contact and feedback (6) expectation setting and ground rules (7) culture of PPI (8) surprise, underestimation and self-selection (9) reciprocity, incentivisation and participation. Process factors: (10) practicalities, timing and barriers (11) parental role (12) event structure. Descriptive summaries, illustrative quotes and the contributors are summarised in Table 2 (context) and Table 3 (process).

**Table 2:** Descriptive Summaries and Illustrative Quotes (context factors)

Descriptive Summary		llustrative Quote		Ppt.
CONTEXT	(1) CCRF ‘role’ perspective	(1a)	“I think my focus of my CCRF work is slightly different because obviously it’s the end, more of the end result of the facility being hired out and patient experience there”	S02
		(1b)	“[the Children’s Board] just felt like another thing I was doing because I did [a study]”	C02
	(2) Purpose, remit and future direction	(2a)	“I think that is what we have done, when you are here, we have to look after you, you tell us what you want, and that’s what we have used it for”	S03
		(2b)	“I think it was about getting a child’s perspective of being involved in research and what they understood by it, and what was good and bad about it and what could be improved”	P02
		(2c)	“At the moment they don’t have a purpose now because they’ve done the information booklets for the CCRF, we’ve got the information that we asked from them from the ‘Big Event’ … but actually there is nothing else for them to do, at the moment”	S01
	(3) Aspirations	(3a)	“I personally think we should be involving them to improve their experience of our physical facilities, educating them about research in general and participation and the whole ethics and everything that goes with it, encouraging their participation in research and potentially leading to them wanting careers in research”	S04
		(3b)	“Yes because it is showing us what we would read, not like what older people would want to read, but what we would want … because like sometimes it is too much information”	C02
	(4) Learning as reciprocation	(4a)	“I think an educational element, rather than us just taking, that we give something back as well”	S03
		(4b)	“Maybe a bit of that [education] but I felt the day was more about learning from the children rather than teaching the children”	P02
		(4c)	“[more education?] No, I think that it was fine the way it was”	C02
	(5) Regular meetings, contact & feedback	(5a)	“It would be nice for the children to see that their comments made a difference, show them the revamped patient information leaflet”	S02
		(5b)	“You would come back in and be like, right what did we do last time”	C02
	(6) Expectation setting & ground rules	(6a)	“I don’t think you should necessarily set too much as expectations, but maybe set what, it’s a good question, what we would like to see, what we want from you as opposed to what you have to give us … maybe what we are trying to achieve rather than you need to turn up to”	S03
		(6b)	“You don’t want to make it like a rulebook like you can’t do this, you can’t do that, you can’t do this, you can’t do that, it’s too much like school no, then they won’t want to do it”	C02
	(7) Culture of PPI	(7a)	“I think the current purpose is, it looks good, if I’m being totally honest, it does look good that we are doing our bit for PPI”	S01
		(7b)	“I thought it was really good that they wanted to know what children thought and didn’t just use an adult assumed version of what children want”	P02
	(8) Surprise, underestimation & self-selection	(8a)	“I was surprised, really, really surprised at the level of knowledge the children had, I had underestimated what they were capable of doing”	S02
		(8b)	“I know these people are self-selecting, they’ve got to be self-selecting, you don’t just come across a hospital find a poster and go oh, that’s a really good thing, they are probably interested in research anyway”	S03
		(8c)	“Because I like to help out”	C01
	(9) Reciprocity, incentivisation & participation	(9a)	“I don’t equally think they should be paid really, I think a thank you but a small thank you”	S01
		(9b)	“We also get the clipboard and all those things”	C02
		(9c)	“Yes, I think I would but I think it just makes it a little bit nicer”	C02
		(9d)	“I think now the sort of onus on children to do extra-curricular activities for their CVs is quite important … we gave them a certificate so it will be good for their CV and it would be good if they do on going work that they can talk about when they go to interviews and things”	S02

Abbreviation: *ppt* = participant

**Table 3:** Descriptive Summaries and Illustrative Quotes (process factors)

Descriptive Summary		Illustrative Quote		Ppt.
PROCESS	(10) practicalities, timing & barriers	(10a)	“Involving kids is also something, I mean do you involve them in school hours, not in school hours, do you involve them at weekends”	S03
		(10b)	“Weekends are better, I don’t want him to miss school in secondary school, after school would be a bit of a push because of where we live but holidays and weekends”	P01
	(11) Parental role	(11a)	“Parents need to be involved in terms of permission to contact them, but that’s probably it I think”	S03
		(11b)	“If you’ve got that child’s mother or father present, they obviously have a greater understanding of their child, say if we explain something in one way that they didn’t quite understand it, the parent might have been able to explain it in a different way that could have got you more of a response”	S05
		(11c)	“The parents do influence the children’s behaviour, I am sure of it, and I think obviously we want pure children’s perception and opinions and it’s difficult to get that if there is a parent, or there is an adult nudging them”	S04
		(11d)	“I think it was good because then it made you comfortable with everyone in the room and then you were comfortable, so then you felt safe because of course your parents were there, then once they were there for the first two it was ok then leaving you for the other ones”	C02
	(12) Event structure	(12a)	“If I pushed them to do everything that was on the list, I don’t think that we would have got quite as much quality out of it”	S03

## Context of PPI

### (1) CCRF ‘role’ perspective

The CCRF facilitates or hosts research through the provision of specialised facilities, support and equipment [16]. In practice this means research projects are at the data collection phase when they come to the CCRF. Most of the staff participants (3/5) perceived this late stage involvement context to narrow remit (see Table 2, 1a). The CCRF role perspective translated differently for parents and CYP, for them (4/4), their Children’s Board and research participation experiences were conflated (see Table 2, 1b).

### (2) Purpose, Remit & Future Direction

Staff (5/5), parents (2/2) and CYP (1/2) expressed fairly unified beliefs about the purpose and remit of the Children’s Board at conception (Table 2, 2a and b). Staff seemed particularly concerned about continued purpose moving forward (Table 2, 2c). One parent felt that meetings should be driven by need and purpose for the CYP focussed on the tasks they performed during meetings (e.g. room redesign, toy purchase).

### (3) Aspirations

Despite the perception of context as a limiting factor, all staff participants (5/5) expressed broader aspirations (Table 2, 3a). When CYP were asked about reviewing information sheets, both (2/2) agreed this was an appropriate ‘job’ for the Children’s Board and might lead to more suitably tailored information (Table 2, 3b).

### (4) Learning as reciprocation

The form of reciprocation most commonly advocated by staff (4/5) was education (Table 2, 4a). One parent felt that the focus of the event should be to learn from CYP, not educate them and the young person that could be drawn on this enquiry felt a larger education focus was not required (Table 2, 4c).

### (5) Regular meetings, contact and feedback

Staff (3/5) often expressed disappointment at the level of feedback and regularity of contact achieved (Table 2, 5a). Many felt regular contact and feedback were important for motivation, continued participation and satisfaction. For one young person, dormant periods between meetings did impact on continuity (see Table 2, 5b).

### (6) Expectation setting and ground rules

For both staff (5/5) and CYP (2/2) desire to hold more frequent meetings and establish a more regular system of feedback did not translate into defined roles for board members with a charter of participation (Table 2, 6a & 6b).

### (7) Culture of PPI

Discussions on remit and direction on occasion revealed scepticism about motivations amongst staff (3/5) (Table 2, 7a). Parents (2/2) did not reveal similar beliefs (Table 2, 7b), and this topic was not established in the CYP interviews.

### (8) Surprise, Underestimation & Self-Selection

Staff (4/5) consistently acknowledged underestimating young people’s capabilities and these statements (3/5) were often tempered with comments on the characteristics of the participants as not representative (Table 2, 8a & 8b). Parents (2/2) and CYP (2/2) on the other hand, attributed participation to altruism (see Table 2, 8c).

### (9) Reciprocity, Incentivisation and participation

Staff unanimously agreed that reciprocation or reward were important factors (5/5). There was no consensus on financial rewards (Table 2, 9a) and varying methods of reimbursement were trialled over the lifespan of the Children’s Board. As a minimum, all participants were provided with refreshments, ‘goody bags’ and certificates. In one interview the parent and young person fondly recalled the goody bags. When asked they confirmed they would have attended regardless of reward but that acknowledgement was ‘nice’ (Table 2, 9c). Position on reward was not established in the other CYP/parent interview. No staff members expressed discomfort with financial rewards however they did suggest alternatives such as personal development (Table 2, 9d).

## Process of PPI

### (10) Practicalities, timing and barriers

Aspirations regarding the possible scope of the Children’s Board were not only perceived by staff to be limited by the CCRFs purpose, but by practical aspects (5/5). There was convergent thinking from parents (2/2) in terms of practicalities and timings (see Table 3, 10a &10b).

### (11) Parental role

For staff, parental involvement encompassed three concepts: parents as gatekeepers for consent (3/5 staff, 1/2 parents) (Table 3, 11a); parents as motivators (2/5 staff) (Table 3, 11b) and parents as regulators of how their child participates (4/5 staff 1/2 parents) (Table 3, 11c). Interviews with CYP revealed another strand to this concept, parent as reassurance (Table 3, 11d).

### (12) Event structure

All Children’s Board events were relatively rigidly planned by staff; however on reflection not all staff (3/5) felt this was necessarily the right approach (Table 3, 12a). Parents and CYP views on this were not established.

## Discussion

### CCRF ‘role’ perspective / aspirations

From the interviews it would seem potential impacts of the Board were limited by the perception staff had of the CCRFs ‘role’. Staff believed there to be appropriate activities the CCRF could and should engage in, that was based on the CCRFs remit of provider of facilities, staff and equipment to other researchers. For example, the assessment of unit equipment by Children’s Board members was gauged fitting, whereas the review of the wider paediatric research community’s participant information sheets was not. Interviewees described ‘late’ involvement in the clinical research process (e.g. post regulatory and governance approvals) which meant the opportunity to engage in many typical PPI activities like review of patient information sheets and protocols, was perceived to have passed.

It could be argued this is a problem of perception as many researchers seeking to use the CCRF make enquiries at the funding application stage. There was therefore potential for the Children’s Board to be flagged as a resource to researchers at this point, which may have led to broader opportunities like review of information sheets, one of the aspirations identified. Previous research has highlighted the importance of ‘role’ within PPI efforts. Evans et al. [5] termed this factor the ‘field of research’ and described how this ‘structured the opportunities and boundaries’ for involvement and Howe et al. [2] similarly identified the ‘boundaries of different roles’. On the basis of these and previous findings it is clear exploring ‘role’ early on is vital. In the CCRF’s case, earlier mapping of opportunities or canvassing/involving staff external to the CCRF may have helped realise these opportunities sooner.

### Purpose, remit and future direction

Staff perspective of purpose, remit and future direction of the Children’s Board were similarly influenced by role and day to day processes on the CCRF. Staff described the transient CCRF/participant relationship, whereby CCRF involvement ceases the moment the participant exits the physical premises and the implications of this on continuity of relationships. Continuity of relationships was acknowledged by one young person however the context was less negative than the staff perception. Remit also appeared to be influenced by the spontaneous nature of the boards development which meant that at conception, there was no collective discussion of purpose, or acknowledgement of whether the Board, as it arose, was what Wilson et al. [6] describes as a ‘one-off model’ or a long-term ‘entwined model’ of PPI. Shippee et al. [24] have previously identified clarity of role and mutual understanding of goals as important components of successful PPI and Gibson et al. [25] state developing a clear idea about desired achievements will determine the type and level of involvement. Brett et al. [1] see defined roles as part of the essential architecture of PPI and Howe et al. [2] identify clarity and review of role as key ingredients for good PPI. On reflection, it could be argued that the Children’s Board method was not an appropriate way to operationalize involvement as it carries the expectation of regular meetings, on-going remit and a continuous relationship. An alternative would be Brady’s ‘Hub and Spoke’ model [14] which entails having a group of core on-going young advisors, alongside one to one, small group and one-off consultations.

### Learning as reciprocation

Development of new skills and knowledge was an anticipated theme as previous studies have identified these as enabling factors [1, 24, 26]. Specific research training was not identified from interviews and development of workplace skills, a benefit perceived by staff, had not occurred to the CYP or their parents. For staff, research education was perceived to be very important and was entwined with reciprocation. This was not replicated in the CYP or parent interviews. This may be a significant factor for PPI with CYP requiring further exploration, as it could represent a misunderstanding of children’s desires in relation to PPI, or the application of the views of a minority to the majority. It is worth positing this represents an example of unconscious bias regarding the knowledge capabilities of CYP; that they need to be ‘educated’, rather than recognised as equal partners. Kellet et al. [27] has previously postulated that a perception that children do not have sufficient knowledge and understanding can be a significant barrier to meaningful participation.

### Surprise, underestimation and self-selection

Subjective judgement of competence is evident from the examples of surprise and underestimation in staff interviews. Although sincere, such comments probably reflect age-related perceptions of knowledge limitations and the power imbalance between adults and CYP and healthcare providers and patients [28]. Developing awareness of unconscious bias and challenging traditional power relationships may improve the facilitation of, or encourage a broader scope to, PPI initiatives. Evans et al. [5] similarly describe surprise amongst research professionals at the level of enthusiasm in their case studies. Wilson et al. [6] suggest reductive perceptions of lay capabilities ‘limited involvement’ and Brett et al. [1] detailed frustrations at ‘assumed lack of knowledge’. These findings imply research professionals need not be so tentative in their approach to involvement and give researchers confidence in bringing complex issues to the table. Perceptions of staff on the ‘self-selecting’ nature of the CYP are interesting, and if demonstrated, may relate to what Martin et al. [29] have termed ‘representative legitimacy’. There was no perception of self-selection amongst the CYP and parents however. In this respect our findings are similar to that of Parson’s et al. [30], who determined altruism to be the main motivator for both research and PPI participation.

### Reciprocity, incentivisation and participation

Education was the staff members preferred method of recognizing contribution; parents expressed no clear message and the CYP described as much satisfaction from a lanyard as with a voucher. Within the PPI literature findings on incentives have been varied. Wilson et al. [6] found divergent opinions in their case studies, with some contributors linking payment to professionalization and others seeing it as standard practice. Mawn et al. [17] have explored incentives with CYP and described how other factors (personal skills) were more important than vouchers and payments. Kirby’s 2004 PPI guide [31] states participation ‘can add to young people’s CVs’. Evans et al. [5] reported career development was viewed as a positive outcome of involvement for CYP. Goodman et al. [32] conducted interviews with the young scientist on their project and found they had used the experience to ‘bolster their CVs’. Lastly Parsons et al. [30] found some CYP thought compensation could lead to the participation of those ‘who do not feel strongly about the research’. Recent guidance documents [33] and top tips [12] recommend reimbursing out of pocket expenses as a minimum and open discussion to establish potential and preferred methods to recognise contributions, recommendations our interviews would endorse.

### Flexibility versus rigour in PPI

Previous research has identified feedback to be an important factor for establishing positive relationships, motivation and impact [1, 5–7]. For staff, this was an important factor; for parents and children this seemed less critical however it did potentially impact on continuity and efficiency. Although more regular contact and feedback were noted ambitions, the data did not suggest participants sought methods to formalise this (for example having a Charter of Participation) and children were not supportive of a formalised approach. This finding reflects that of Evans et al. [5] who explored ‘defined roles’ in relation to impact and concluded formal role definition was not necessary, rather it was more helpful for researchers to make clear their practical expectations. Guidelines from the Public Health, Education, Awareness, Research (PEAR) [34] project published in 2010 recommend agreeing clearly defined roles. Similarly, Howe et al. [2] established making clear what is expected is important for meaningful involvement and Hawke et al. [10] contend a shared, clear understanding of role is essential. This potentially contradictory position, desire for clarity of role yet rejection of formalised contracts or agreements could be problematic and should be openly discussed and agreed early in any PPI endeavour.

### Culture of PPI

In staff interviews questions on remit and role on occasion revealed cynicism about motivations. Evans et al. [5] have established organisational culture and values towards PPI to be an important contextual factor for effective public involvement in research. Wilson et al. [6] also found a clear link between what they term ‘value sets’ and the construction, operationalization and evaluation of PPI endeavours and Brett et al. [1] also detailed pervasive tokenistic attitudes towards PPI. It could be argued underlying scepticism towards PPI as ‘looking good’ (S01) or ‘meeting a metric’ (S03) has shaped views regarding motivation and possibly stymied development of the board’s remit. However, this position is not fully supported by the data and the more influencing factor on purpose and remit would appear to be the perception that the research structure on the CCRF (context) was not compatible with the board format (mechanism).

### Practicalities, timing and barriers, event structure

Practicalities and structure of meetings and the parental role were the three process aspects identified in the content analysis. The challenge of arranging meetings was very clear in all interviews; however is not specific to the board format. Across all their case studies Evans et al. [5] found organisation to be difficult. Mawn et al. [13] has previously demonstrated the busy lives of CYP can be a barrier to involvement and it is essential to arrange meetings around other commitments. Brady et al. [14] found that a dynamic and flexible approach in tune with the ‘rhythms, preferences and commitments’ of young people’s lives was required. This is perhaps where a charter of participation may become useful as it makes clear the level of commitment or flexibility. To ensure meetings were engaging staff often went to considerable lengths to plan them. Some staff reflected rigidly structured programmes could be constraining, however everyone recalled them as being fun. Brett et al. [35] have previously found poor planning can result in negative attitudes, lack of trust and less chance of beneficial impact. It seems reasonable to conclude that finding the balance between planning and flexibility is vital.

### Parental roles

Parental involvement was discussed in three formats by staff and parents (gatekeepers, motivators, regulators) and in terms of reassurance by CYP. Parents were acknowledged to be the gatekeepers to involving CYP and parental permissions were taken very seriously. This was never perceived to be burdensome or a barrier, as has been the case for other initiatives with CYP [34]. The staff interviews determine the preferred extent of parental involvement was as gatekeeper; however the role of parents in terms of being a motivator to participation (encouragement, confidence booster, and explainer) and as regulator (monitor of behaviour and participation) were also recognised. Most staff members believed parental presence regulated the open participation of CYP (Table 3, 11c) and there was frequent reference to the importance of obtaining the CYP voice. When discussing meetings and reflecting on parental involvement however, no one explicitly stated Board proceedings were affected by parental presence. Evans et al. [5] describe a ‘level of informality’ as important for constructive dialogue, something which may not be achievable in the presence of parents, especially if they do act as ‘regulators’. Mawn et al. [13] found formality to be a barrier which can ‘prevent involvement and reduce productivity’. Parental involvement in the early Children’s Board sessions may have produced formality and regulated participation; however the actual effect or specific examples is not explicit in the data. It is interesting that the CYP found parental presence preferable in the formative stages of PPI work.

### Reflections/critical perspective

There are significant limitations to this project. This was not a collaborative evaluation; no CYP were co-researchers, therefore potentially important questions could have been missed. The sample size is extremely small, which limits the findings significantly, especially in relation to CYP as there were only two participants. Demographic data was not collected so the ‘representative legitimacy’ component highlighted in staff interviews, could not be interrogated. Moreover, no theoretical frameworks were employed which may have aided interpretation. Participants were retrospectively reviewing past experiences (Children’s Board meeting were held in January 2013, June 2014 and September 2015 and interviews conducted between August 2015 and December 2017); therefore responses are subject to recall bias. Additionally, staff members were interviewed by their peer therefore responses may be influenced by social desirability bias and/or staff may have felt reluctant to speak critically for fear it may have affected their employment. Similarly CYP were interviewed with their parents, which could have affected their responses. Conversely, all participants may arguably be motivated to speak positively of any initiative in which they are involved.

### Implications and conclusion

The most significant finding of our interviews relates to the appropriateness of the mechanism of involvement in relation to context for involvement. Advisory groups, boards or panels are a common mechanism of implementing involvement, most likely as they are a relatively inexpensive and convenient way to involve people in research. However our data would suggest that significant consideration should be given before selecting this format when involving CYP for although it may initially satisfy objectives, it is a relatively static approach that over time may not suit the context. In this case, the staff interviewed felt the role of the CCRF as host or facilitator limited the opportunities for involvement, and the board mechanism that has connotations of a frequent meeting schedule, quickly meant the remit was exhausted. Prior acknowledgement and consideration of the context, opportunity mapping and wider stakeholder engagement may help improve the implementation of future involvement activities. Other context and process factors identified echo that of previous studies. Further research is required to explore the importance of education and career development as ‘impacts’ of PPI with CYP and the age and/or professional status power differential facilitators (in this case healthcare staff) may bring to the arena. Lastly, consideration should also be given to moving beyond simple descriptions of impact [8], for although tangible outputs were described in this study; an embedded evaluation methodology would have enabled assessment of real world meaningfulness and more robust measurement.

## Abbreviations

CCRF: Cambridge Clinical Research Facility
GRIPP2: Guidance for Reporting Involvement of Patients and the Public (GRIPP2) checklist
NIHR: National Institute for Health Research
PEAR: Public Health, Education, Awareness, Research
PPI: Patient and Public Involvement
YPAG: Young Person’s Advisory Group
